# STIM1-mediated bidirectional regulation of Ca^2+^ entry through voltage-gated calcium channels (VGCC) and calcium-release activated channels (CRAC)

**DOI:** 10.3389/fncel.2014.00043

**Published:** 2014-02-24

**Authors:** Osama F. Harraz, Christophe Altier

**Affiliations:** ^1^Department of Physiology and Pharmacology, Hotchkiss Brain Institute, Libin Cardiovascular Institute, University of CalgaryCalgary, AB, Canada; ^2^Department of Pharmacology and Toxicology, Faculty of Pharmacy, Alexandria UniversityAlexandria, Egypt; ^3^Department of Physiology and Pharmacology, Snyder Institute for Chronic Diseases, Inflammation Research Network, University of CalgaryCalgary, AB, Canada

**Keywords:** STIM1, Orai, VGCC, CRAC, L-type, T-type, calcium channels, store-operated Ca^2+^ entry

## Abstract

The spatial and temporal regulation of cellular calcium signals is modulated via two main Ca^2+^ entry routes. Voltage-gated Ca^2+^ channels (VGCC) and Ca^2+^-release activated channels (CRAC) enable Ca^2+^ flow into electrically excitable and non-excitable cells, respectively. VGCC are well characterized transducers of electrical activity that allow Ca^2+^ signaling into the cell in response to action potentials or subthreshold depolarizing stimuli. The identification of STromal Interaction Molecule (STIM) and Orai proteins has provided significant insights into the understanding of CRAC function and regulation. This review will summarize the current state of knowledge of STIM-Orai interaction and their contribution to cellular Ca^2+^ handling mechanisms. We will then discuss the bidirectional actions of STIM1 on VGCC and CRAC. In contrast to the stimulatory role of STIM1 on Orai channel activity that facilitates Ca^2+^ entry, recent reports indicated the ability of STIM1 to suppress VGCC activity. This new concept changes our traditional understanding of Ca^2+^ handling mechanisms and highlights the existence of dynamically regulated signaling complexes of surface expressed ion channels and intracellular store membrane-embedded Ca^2+^ sensors. Overall, STIM1 is emerging as a new class of regulatory proteins that fine-tunes Ca^2+^ entry in response to endoplasmic/sarcoplasmic reticulum stress.

## Ca^2+^ handling mechanisms

The second messenger calcium (Ca^2+^) plays a crucial role in a broad range of eukaryotic cellular functions. Regulation of its intracellular concentration ([Ca^2+^]_*i*_) represents a major determinant that controls signal transduction pathways such as secretion, excitation/contraction coupling, motility, transcription, growth, cell division or apoptosis (Berridge et al., 2003; Catterall, 2011). Precise neural circuit formation and control of neuronal excitability necessitate the tight handling of Ca^2+^. Further, Ca^2+^ signals are crucial for synaptic transmission and plasticity (Berridge, 1998). In addition, pathophysiological neural insults such as cerebral ischemia can evoke an unwanted rise in Ca^2+^ leading to Ca^2+^ overload toxicity and neuronal cell death (Berridge, 1998; Arundine and Tymianski, 2004).

**Figure 1:**
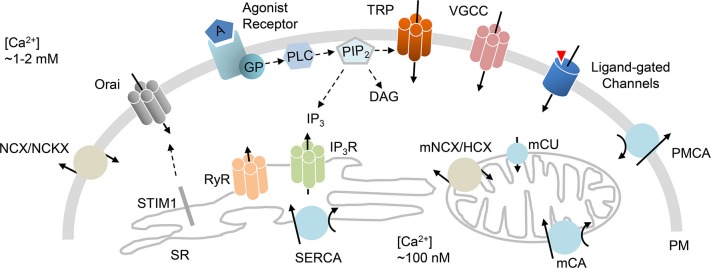
**Ca^2+^ handling mechanisms.** Schematic diagram that highlights ion channels and transporters directly implicated in Ca^2+^ homeostasis. Influx of Ca^2+^ is primarily mediated by VGCC, receptor-mediated Ca^2+^ entry, transient receptor potential channels (TRP), ligand-gated channels, and store-operated Orai channels that are activated by STIM1 protein. Efflux of Ca^2+^ is achieved by PM Ca^2+^ ATPase (PMCA), Na^+^/Ca^2+^ exchanger (NCX) or Na^+^/Ca^2+^/K^+^ exchanger (NCKX). Release of Ca^2+^ from the ER/SR is mediated through IP_3_ (IP_3_R) or ryanodine (RyR) receptors. The reuptake of Ca^2+^ into the ER/SR is primarily mediated by sarcoplasmic/ER Ca^2+^ ATPase (SERCA). Mitochondrial Ca^2+^ handling incorporates mitochondrial Ca^2+^ uniporter (mCU), Ca^2+^ ATPase (mCA) or Na^+^/Ca^2+^ or H^+^/Ca^2+^ exchangers (mNCX, mHCX). GP (G proteins); PIP_2_ (phosphatidylinositol 4,5-bisphosphate); PLC (phospholipase C).

Strict handling of intracellular Ca^2+^ is necessary to maintain optimized cellular functions. In general, Ca^2+^ signals are modified by the control of Ca^2+^ flux in (entry) and out (efflux) of the cell through plasma membrane (PM) channels and transporters that facilitate Ca^2+^ movement between the extracellular milieu and cytoplasm across a Ca^2+^ concentration gradient (Berridge et al., 2003). In addition, integral proteins localized in the membranes of intracellular stores allow Ca^2+^ release (to the cytoplasm) and reuptake (into the Ca^2+^ store). The main intracellular Ca^2+^ stores are the endoplasmic reticulum (ER) and sarcoplasmic reticulum (SR). A number of regulatory mechanisms have been proposed to mediate the cellular influx, efflux, release and reuptake of Ca^2+^, thus achieving Ca^2+^ homeostasis within the cell (Berridge et al., 2003; Stutzmann and Mattson, 2011). Accumulated data suggest that this homeostasis involves the concerted action of Ca^2+^ entry channels at the PM and Ca^2+^ release channels in intracellular ER/SR stores (Figure 1).

Over the past decades, it has been recognized that Ca^2+^ influx into neuronal subcellular compartments (e.g., dendrites, somata, spines, axons) is mediated by two principal means of Ca^2+^ entry. These routes are voltage-gated Ca^2+^ channels (VGCC) and ionotropic neurotransmitter receptors (Berridge, 1998; Catterall, 2011), both routes elicit crucial rises in cytosolic Ca^2+^ in response to different stimuli. VGCC are widely expressed in excitable cells and they trigger Ca^2+^ influx over specific ranges of membrane potentials. Activation of VGCC generates fast neurotransmission at nerve terminals (Bezprozvanny et al., 1995), or excitation-contraction coupling in cardiac, skeletal and smooth muscle cells (Catterall, 2011; Tuluc and Flucher, 2011; Navedo and Santana, 2013). Neurons along with other cell types display an alternative Ca^2+^ entry mode that is coupled to intracellular Ca^2+ ^ stores (Gemes et al., 2011). This alternative type of entry, known as capacitative calcium entry, is triggered upon the depletion of Ca^2+^ stores to facilitate store-operated Ca^2+^ entry (SOCE). The latter, SOCE, would in turn replenish the intracellular ER/SR stores (Soboloff et al., 2012). Extensive work on this route of calcium influx has established its functional importance in neurons and its ability to supplement cytosolic Ca^2+^ required for neurotransmission (Berna-Erro et al., 2009; Gemes et al., 2011).

## store-operated Ca^2+^ entry (SOCE): a still-developing story

About three decades ago, Putney first described the concept of capacitative Ca^2+^ entry (Putney, 1986). According to this concept, the concerted control of both Ca^2+^ influx and Ca^2+^ release from intracellular stores orchestrates Ca^2+^ homeostasis. In other words, Ca^2+^ influx is modulated by the capacity of the cell to hold Ca^2+^. Several studies showed that stimulus-evoked ER/SR depletion can trigger subsequent influx of extracellular Ca^2+^ into the cytoplasm as a means to replenish Ca^2+^ in intracellular stores (Takemura and Putney, 1989; Muallem et al., 1990). These findings led Putney’s model to be revised by indicating that the activation of PM Ca^2+^ channels was a direct consequence of ER/SR depletion (Putney, 1990). Entry of extracellular Ca^2+^ upon store depletion was later suggested to be mediated by Ca^2+^-release activated channels (CRAC) in a process referred to as SOCE (Hoth and Penner, 1992; Patterson et al., 1999).

**Figure 2:**
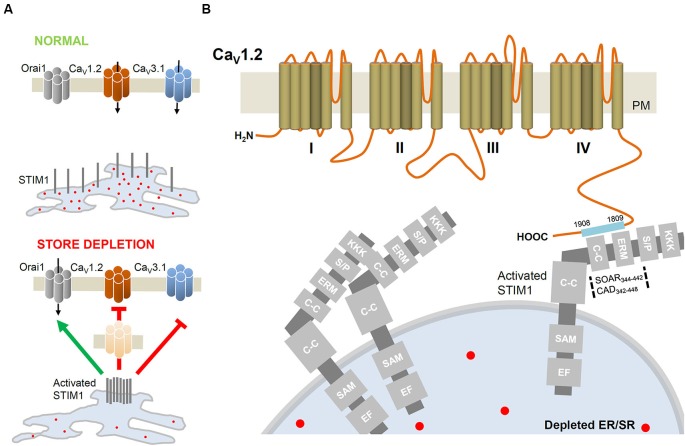
**STIM1-mediated regulation of VGCC. (A)** In basal conditions, the intraluminal EF-hand of STIM1 is occupied by Ca^2+^. Upon ER/SR depletion, STIM1 molecules aggregate closer to PM to activate Orai channels but inhibit Ca_V_1.2 (L-type; Park et al., 2010; Wang et al., 2010) and Ca_V_3.1 (T-type; Nguyen et al., 2013) channels. STIM1-induced internalization of Ca_V_1.2 removes functional channels from the cell surface. **(B)** Detailed interacting sites of STIM1 and the C-terminus of Ca_V_1.2. The STIM1 segments CAD (342–448; Park et al., 2010) or SOAR (344–442; Wang et al., 2010) interacts with the C-terminus (1809–1908; Park et al., 2010) of Ca_V_1.2. C-C, coiled-coil domain; EF, EF hand motif; ERM, Ezrin-Radixin-Moesin domain; KKK, lysine rich domain; SAM, sterile-α motif; S/P, serine/proline rich domain.

The CRAC was first described in 1992 (Hoth and Penner, 1992), but its mechanism of activation was not revealed until 2005 when Cahalan and coworkers identified the STromal Interaction Molecule 1 (STIM1) as the intracellular CRAC component that acts as the Ca^2+^ sensor. Upon store depletion, this sensor STIM1 aggregates and activates the PM Ca^2+^ channel that is necessary for SOCE (Roos et al., 2005; Zhang et al., 2005). First physiological description of STIM1 as a key component of CRAC was in *Drosophila* S2 cells in which SOCE is the predominant Ca^2+^ entry mechanism. Using RNA interference screens of candidate genes, they reported that *Stim* loss altered SOCE (Roos et al., 2005; Zhang et al., 2005). It was 1 year after the intracellular STIM1 was discovered that the PM component of CRAC was identified. The *Orai* gene, named after the mythological keepers of heaven’s gate, was determined as a result of genetic mapping of mutations linked to impaired lymphocyte function (Zhang et al., 2006). The SOCE mechanism was then revised to involve the two key players: (1) STIM, a transmembrane Ca^2+ ^ sensor protein that is primarily embedded into the SR/ER membrane; and (2) Orai, an integral PM protein being the pore-forming subunit of the CRAC channel (Figures 1, 2A; Soboloff et al., 2012).

Once the key genes governing SOCE were identified, the interplay between STIM1 and Orai was extensively examined. Investigators reported that Orai protein monomers multimerize to form a Ca^2+^ channel whose activity is triggered by interaction with STIM1 (Penna et al., 2008). Further, STIM-Orai Activating Region (SOAR) and CRAC Activation Domain (CAD) were identified as active STIM1 sites necessary to trigger the CRAC current (Park et al., 2009; Yuan et al., 2009). In addition, high-resolution crystal structures of the CAD and the N-terminal region of STIM1 as well as the full-length Orai channel were recently characterized (Stathopulos et al., 2008; Hou et al., 2012; Yang et al., 2012). These latter discoveries represent major landmarks towards the elucidation of the conformational changes of STIM1-Orai complexes as well as the possible interactions with key proteins involved in Ca^2+^ handling mechanisms.

## STromal Interaction Molecule (STIM): the Ca^2+^ sensor controlling Ca^2+^ entry

STIM1 was first reported as a growth modulator (Oritani and Kincade, 1996), and was not implicated in cellular Ca^2+^ dynamics until 2005 (Roos et al., 2005). It became evident that STIM1 proteins function as Ca^2+^-sensing molecules, resident in the membranes of the intracellular Ca^2+^ stores (ER/SR), that regulate SOCE (Soboloff et al., 2012). STIM1 molecule is ubiquitously expressed and is involved in a wide range of cellular functions. It is an essential component of SOCE in lymphocytes (Liou et al., 2005; Zhang et al., 2005), platelets (Varga-Szabo et al., 2008), neurons (Berna-Erro et al., 2009; Venkiteswaran and Hasan, 2009; Gemes et al., 2011), skeletal muscle cells (Stiber et al., 2008), and cardiomyocytes (Touchberry et al., 2011). When Ca^2+^ stores are depleted, STIM1 molecules aggregate and activate the Orai channels to facilitate Ca^2+^ influx (Soboloff et al., 2012).

The STIM2 was identified as a homologue of STIM1. Both isoforms are ubiquitously expressed in vertebrates and the expression level of STIM1 is generally higher than STIM2 in most tissues (Williams et al., 2001; Oh-Hora et al., 2008). Notably, STIM2 is primarily found in dendritic cells (Bandyopadhyay et al., 2011) and the brain (Williams et al., 2001; Berna-Erro et al., 2009). The two proteins conserve high homology region, but their C- and N-termini display substantial divergence (Soboloff et al., 2012). Interestingly, structural aspects of the two proteins engender subtle differences that are associated with significant functional implications. Although primarily localized to the intracellular ER/SR membranes, approximately 10% of STIM1 proteins are integrated in the PM. This is in stark difference to STIM2 which, due to an ER-retention sequence in its C-terminus, is exclusively localized in the ER membrane (Soboloff et al., 2006; Saitoh et al., 2011). In addition, STIM1 is a stronger activator of Orai channels when compared to STIM2 (Bird et al., 2009) and STIM2 is more sensitive to small changes in [Ca^2+^]. These properties of STIM2 and its poor coupling to Orai channels could in theory be essential to limit uncontrolled SOCE (Soboloff et al., 2012). In the following section, our discussion will focus on how STIM1 modulates different Ca^2+^ influx routes.

## STromal Interaction Molecule 1 (STIM1) and Ca^2+^ entry

There are two main stimulus modalities that elicit Ca^2+^ entry: the membrane depolarization in excitable cells versus the ER/SR calcium depletion in non-excitable cells. The VGCC differ from CRAC in being activated by depolarization in response to action potentials or subthreshold stimuli. The STIM1/Orai CRAC complex is activated in response to ER/SR calcium depletion. Noteworthy, both channels are expressed in excitable and non-excitable cells (Kotturi and Jefferies, 2005; Lyfenko and Dirksen, 2008; Stiber et al., 2008). However, VGCC predominate in excitable cells (e.g., neurons, cardiomyocytes, smooth muscle cells) while CRAC currents are prevalent in non-excitable cells (e.g., T-lymphocytes). Both Ca^2+^ channels have received significant attention. In this review we will focus our discussion on the mechanisms through which STIM1 interaction with CRAC and VGCC modulate Ca^2+^ influx.

### STromal Interaction Molecule 1 (STIM1) stimulates Ca^2+^-release activated channels (CRAC)

In a resting cell, STIM1 exhibits a tubular distribution throughout the ER/SR (Roos et al., 2005). The N-terminus of STIM1 resides inside the ER/SR lumen and possesses an EF-hand that binds Ca^2+^ with low affinity (200–600 nM), and thus acts as a Ca^2+^ sensor. When the lumen of the ER/SR is full of Ca^2+^, STIM1 EF-hands are saturated with Ca^2+^ ions. In contrast, upon stores depletion, STIM1 molecules aggregate into oligomers (Soboloff et al., 2012) and translocate to sites where the ER/SR membrane is closer to the PM. In these microdomains, STIM1 oligomers form clusters which interact with and activate Orai channels (Figure 2A; Zhang et al., 2006; Penna et al., 2008; Soboloff et al., 2012). STIM1 activates Orai1 by the region identified as STIM1-Orai activating region (SOAR) or CAD to facilitate Ca^2+^ influx. Noteworthy, the small portion of the STIM1 pool integrated into the PM is not required for CRAC channel activation (Park et al., 2009; Yuan et al., 2009).

### STromal Interaction Molecule 1 (STIM1) inhibits voltage-gated Ca^2+^ channels

SOCE upon ER/SR depletion has been extensively studied since first proposed. Recently, another mechanism of calcium influx was found to be suppressed by store depletion (Park et al., 2010; Wang et al., 2010). This led to the novel term “store-inhibited channels (SIC)” (Moreno and Vaca, 2011); in contrast to store-operated channels that activate upon Ca^2+^ store depletion (Soboloff et al., 2012). The first class of channels identified to be inhibited by store depletion is the voltage-gated Ca^2+^ channel.

#### STromal Interaction Molecule 1 (STIM1) inhibits Ca_V_1.2 channels

The predominant expression and function of CRAC in non-excitable cells is well reported. The key components of CRAC, STIM and Orai, are also expressed in excitable cells (Stiber et al., 2008; Berna-Erro et al., 2009; Venkiteswaran and Hasan, 2009; Gemes et al., 2011; Touchberry et al., 2011) where VGCC predominate as the main route of Ca^2+^ entry in response to depolarizing stimuli. One subtype of VGCC, the Ca_V_1.2 channel, is ubiquitously expressed in neuronal, cardiac and smooth muscle cells. The Ca_V_1.2 L-type channel is involved in specific cellular functions and has been long considered as an important target for therapeutic agents such as antiarrhythmic and antihypertensive drugs (Catterall, 2011). In order to understand the coordinated interaction between CRAC and the L-type calcium channel, two studies have examined the role of STIM1 in regulating Ca_V_1.2 and Orai1 function. Interestingly, by employing a divergent array of approaches, these studies reported an inhibitory interaction between STIM1 and Ca_V_1.2 (Figures 2A, B). This functional crosstalk may explain the predominance of either CRAC or VGCC activity in different tissue types (Park et al., 2010; Wang et al., 2010).

Using excitable cortical neurons and vascular smooth muscle cells, Park et al. (2010) and Wang et al. (2010) assessed VGCC and CRAC functions by monitoring cytoplasmic Ca^2+^. Unexpectedly, depletion of ER/SR stores attenuated depolarization-induced Ca_V_1.2 activity. Further, depolarization of non-excitable STIM1-rich T-lymphocytes could not evoke a rise in [Ca^2+^]_*i*_. The modulatory interaction between STIM1 and Ca_V_1.2 was emphasized by the observations that: (1) STIM1 overexpression attenuated Ca_V_1.2 activity; while (2) Ca_V_1.2-mediated responses were enhanced when STIM1 function or expression was impaired. Direct interaction between STIM1 and Ca_V_1.2 proteins was ascertained and a set of experiments, using truncated forms of STIM1, documented that SOAR domain (STIM-Orai activating region, 344–442; Wang et al., 2010) directly interacts with the C-terminus of Ca_V_1.2 α_1_ subunit (1809–1908; Park et al., 2010). Co-immunoprecipitation analysis confirmed the STIM1-Ca_V_1.2 interaction and functional studies revealed that the SOAR domain was necessary and sufficient to suppress Ca_V_1.2 current (Figure 2B). A slower inhibitory interaction was further suggested by Park and coworkers, in which the surface expression of Ca_V_1.2 decreased as a consequence of long-term internalization of the channel from the PM (Figure 2A; Park et al., 2010). Despite having the same core conclusion, the two reports highlight different perspectives. While Park and coworkers proposed an inhibitory mechanism that attenuates channel expression, Wang et al. found a potential role for Orai1 in the STIM1-Ca_V_1.2 inhibitory interaction. Preventing STIM1 expression alone did not abolish Ca_V_1.2 channel suppression while the simultaneous inhibition of both STIM1 and Orai1 was necessary to mask Ca_V_1.2 inhibition by store depletion. In summary, the two groups reported for the first time that STIM1 effectively attenuates Ca_V_1.2 activity.

The description of Ca^2+^ conductances inhibited upon store depletion has major implications for Ca^2+^ signaling in excitable and non-excitable cells. In excitable neuronal cells, VGCC are expressed at higher level than CRAC components and are the predominant Ca^2+^ influx route. In contrast, non-excitable cells display reduced VGCC expression and CRAC represents the main Ca^2+^ entry pathway due to the high expression of STIM1 and Orai (Liou et al., 2005; Zhang et al., 2005; Park et al., 2010). While STIM1 interacts with Orai to facilitate Ca^2+^ influx, a new modulatory role for STIM1 has emerged through which it inhibits Ca^2+^ entry mediated by VGCC. Noteworthy, the ability of STIM1 to reciprocally regulate Orai and Ca_V_1.2 would imply that the mode of action of STIM1 is tissue specific. In other words, STIM1 would typically stimulate CRAC in non-excitable cells and inhibit VGCC in excitable cells (Park et al., 2010; Wang et al., 2010; Moreno and Vaca, 2011). This reciprocal modulation of Ca^2+^ entry by STIM1 seems to play a critical role in Ca^2+^ homeostasis by fine-tuning Ca^2+^ entry in cells that simultaneously express both channel types. By providing this missing piece of evidence, these studies resolved the predominant function of one channel over the other despite their co-expression in excitable and non-excitable tissues. That being said, whether other Ca^2+^ channels exhibit analogous or distinct modulation by STIM1 remains unknown.

#### STromal Interaction Molecule 1 (STIM1) inhibits T-type Ca^2+^ channels

Recent published experiments revealed that Ca_V_1.2 channel is not the only voltage-gated Ca^2+^ channel suppressed by STIM1. Interestingly, a study by Nguyen et al. (2013) showed that STIM1 attenuates the activity of T-type Ca^2+^ channel. Using cardiomyocyte-derived HL-1 cells, an inhibitory association between STIM1 and Ca_V_3.1 was reported and was suggested to limit excessive Ca^2+^ entry. Pathological Ca^2+^ influx in cardiac myocytes can trigger Ca^2+^ overload in the SR and cardiac arrhythmias (Sedej et al., 2010), and STIM1-dependent modulation of Ca_V_3.1 could, in theory, restrain such unwanted entry. Indeed, the authors showed that knocking down STIM1 increases T-type Ca^2+^ current density. They further suggested that STIM1 may regulate the expression of T-type α_1_ subunits as knocking down STIM1 augmented the surface expression of Ca_V_3.1 channel. Whether Ca_V_3.1 trafficking or stability at the PM is implicated remains unknown and requires further investigation. Intriguingly, these findings represent a novel regulatory pathway for Ca^2+^ handling in cardiac myocytes. This regulation may be implicated in the modulation of rhythmicity and excitability of native cardiac pacemaker cells where T-type Ca^2+^ channel expression and function is evident (Mangoni et al., 2006; Nguyen et al., 2013).

## Conclusions

The interaction of STIM1 with CRAC or VGCC extends our understanding of the role of STIM1 in cellular Ca^2+^ handling. Recent published work (Park et al., 2010; Wang et al., 2010; Nguyen et al., 2013) described a novel regulatory function for STIM1. Distinct from its role as an ER/SR Ca^2+^ sensor to facilitate Ca^2+^ entry and replenish the stores, STIM1 suppresses the activity of voltage-gated Ca^2+^ channel in excitable cells. This inhibitory association prevents excessive cellular Ca^2+^ influx by mechanisms including direct protein-protein interaction and reduced VGCC surface expression (Park et al., 2010; Wang et al., 2010; Nguyen et al., 2013). This novel concept raises numerous questions that pertain to the dynamic regulation of cellular Ca^2+^ in health and disease. Since STIM1 bi-directionally regulates VGCC and CRAC, SR/ER stress may lead to pathologies related to downstream Ca^2+^-dependent pathways. In fact, mutations in STIM1 elicit severe immunodeficiency syndromes associated with compromised SOCE (Feske et al., 2010). Impaired STIM-dependent regulation of VGCC and subsequent alterations in Ca^2+^ entry remain to be investigated; as it could be linked to diseases such as epilepsy, cardiac arrhythmia or hypertension. Finally, it will be fundamental to elucidate the cooperated interaction of PM Ca^2+^ channels and their associated subunits and how those signaling complexes respond to ER/SR stress through Ca^2+^ sensors such as STIM1 proteins.

## Author contributions

Osama F. Harraz and Christophe Altier wrote the manuscript. Osama F. Harraz produced the figures.

## Conflict of interest statement

The authors declare that the research was conducted in the absence of any commercial or financial relationships that could be construed as a potential conflict of interest.
